# Reese’s Medical Lexicon

**Published:** 1860-10

**Authors:** 


### Reese's Medical Lexicon.

We learn from the September number of the American Medical Gazette, that a new and enlarged edition of the above convenient little Dictionary is in process of preparation. The forthcoming edition will contain several thousand words more than the one already published, and many not to be found in any other dictionary.

